# Promising Potential of Edaravone‐Loaded Nanogel in Ameliorating of Stroke in Rats: The Modulation of Immune Response

**DOI:** 10.1155/bmri/2275362

**Published:** 2026-09-15

**Authors:** Leila Nasehi, Salar Jahangiri, Soroush Bijani, Mir-Jamal Hosseini

**Affiliations:** ^1^ Department of Medical Laboratory Sciences, School of Allied Medical Sciences, Zanjan University of Medical Sciences, Zanjan, Iran, zums.ac.ir; ^2^ Department of Pharmacology and Toxicology, School of Pharmacy, Zanjan University of Medical Sciences, Zanjan, Iran, zums.ac.ir; ^3^ Zanjan Pharmaceutical Biotechnology Research Center, Zanjan University of Medical Sciences, Zanjan, Iran, zums.ac.ir

**Keywords:** edaravone, *Hsp70*, stroke, *Tlr4*, *Tlr7*

## Abstract

**Objectives:**

Ischemic stroke is mainly caused by the interruption of cerebral blood flow, which causes severe neurological damage and is one of the main drawbacks of death and disability worldwide. Edaravone (EDV) has been clinically used for the treatment of ischemic stroke in Japan and some countries with two major drawbacks: poor water solubility and nephrotoxicity. Therefore, GSH‐PMAA or nanogel was utilized as a drug carrier for EDV to overcome this issue.

**Methods:**

The study was designed to assess the cognitive response and expression of brain tissue genes, including heat‐shock protein (*Hsp70*), Toll‐like receptors (*Tlr4*&*Tlr7*), following treatment with EDV‐loaded nanogel or GSH‐PMAA‐EDV in ischemic rats. Animals were randomly divided into seven groups, and after the induction of the ischemia‐like model, rats received different doses of EDV‐loaded nanogel.

**Results:**

On the day after the novel object recognition test, the rodent hippocampus was dissected and used for the evaluation of gene expression. Real‐time PCR was used to evaluate the gene expression of *Tlr4*, *Tlr7*, and *Hsp70*. These results indicated that after the induction of stroke in animals, the expression of *Tlr4* and *Tlr7* increased significantly. However, no significant change in *Hsp70* expression was observed. However, EDV‐loaded nanogel at doses of 20, 40, and, 250 *μ*g/kg restored the overexpression of *Tlr4* and *Tlr7*.

**Conclusion:**

Treatment with EDV‐loaded nanogel has been shown to improve cognitive behavior and immune response of neural tissue in a transient global ischemic model. These improvements were also achieved at lower equalized doses when compared with the unmodified EDV.

## 1. Introduction

Ischemic stroke continues to represent a substantial worldwide burden of mortality and long‐term disability, accounting for approximately 3.6 million deaths and 7.8 million new cases worldwide in 2021. Although age‐standardized mortality rates have declined substantially since 1990, the absolute global burden of ischemic stroke continues to increase, highlighting the growing need for effective prevention, acute management, and rehabilitation strategies [1, 2]. The distribution of blood supply in the brain is considered more complicated than that in organs with less energy consumption and more space for inflammation. Low blood supply during ischemic stroke leads to an imbalance in ATP formation and ion concentration level. This event results in higher AMPK expression and reduced mTOR activation. As oxidative stress intensifies and mitochondrial function becomes impaired, neural cells upregulate hypoxia‐inducible factor 1*α* (HIF‐1*α*), thereby promoting autophagic responses [3]. Although stroke patients have been extensively investigated by numerous research teams, no exact method for the prevention and treatment of all clinical symptoms has been validated. The latest approach to post‐stroke management consists of thrombolytic, antihypertensive, and glucose‐modulating medications. The utilization of neuroprotective agents within 24 h of the post‐stroke phase promotes axonal guidance and neural regeneration [4]. Many neuroprotective agents have entered the preclinical phase; however, none have entered the clinical or been used for stroke management. Methods such as peritoneal dialysis have shown promising results in suppressing glutamic acid signaling in the brain and further neural death [5].

Oxidative stress is a key mediator of ischemic neuronal damage. Cerebral ischemia and subsequent reperfusion promote excessive ROS and RNS production, leading to lipid and protein oxidation, neuro‐inflammation, mitochondrial dysfunction, and cell death signaling [6, 7]. Excitotoxic NMDA receptor activation promotes intracellular Ca^2+^ accumulation and oxidative stress, thereby exacerbating neuronal injury. Therefore, attenuation of oxidative stress and restoration of endogenous antioxidant defenses are key targets for neuroprotective interventions [5]. Edaravone (EDV) is a low–molecular‐weight antioxidant that has attracted considerable interest as a neuroprotective agent because of its ability to scavenge highly reactive free radicals, including hydroxyl and peroxyl radicals, thereby limiting lipid peroxidation and oxidative stress [8].

Despite its therapeutic potential of EDV and permeability to central nervous system, very short plasma half‐life (5.4 min) may limit sustained drug exposure and necessitate repeated or relatively high‐dose administration which caused potentially increased systemic adverse effects such as nephrotoxicity [9, 10]. Therefore, it is expected that strategies capable of prolonging EDV availability and enhancing its delivery to ischemic brain tissue could improve its neuroprotective potential while potentially reducing the need for high‐dose and repeated administration. Nanotechnology‐based drug‐delivery platforms provide a potential approach to overcoming these challenges by enhancing drug stability, sustaining therapeutic release, and improving targeted distribution to affected tissues. Polymeric nanogels offer several potentially advantageous properties, including high aqueous stability, biocompatibility, high drug‐loading capacity, controlled release, and facile surface functionalization, which may further enhance brain‐targeted delivery and contribute to the neuroprotective effects of this formulation, consistent with our previous findings [11].

In addition to oxidative stress, neuro‐inflammation contributes substantially to secondary neuronal damage following cerebral ischemia [11–13]. Toll‐like receptors (TLRs) recognize endogenous damage‐associated molecular patterns (DAMPs) liberated by neuronal damage and glial cells released from damaged neurons and glial cells and can initiate signaling cascades that regulate neuro‐inflammation, cellular stress, and tissue repair [14, 15]. Among TLRs, TLR4 has been extensively implicated in postischemic neuro‐inflammation and secondary brain injury, whereas the role of TLR7 in ischemic stroke remains comparatively less well characterized [16–18]. Importantly, the effects of TLR signaling are context dependent and different according to receptor subtype, cellular condition, and the temporal stage of ischemic injury [12, 18]. Thus, investigation TLR‐associated pathways may provide mechanistic insight into how antioxidant interventions modulate postischemic inflammation and promote neuronal survival. In addition, HSP70 is a key molecular chaperone that maintains protein homeostasis and regulates cellular stress responses, mitochondrial function, and inflammatory signaling [19, 20].Thus, HSP70 may serve as a molecular link between cellular stress responses, oxidative stress, and neuro‐inflammation following cerebral ischemia [21]. However, the relationship between EDV‐mediated antioxidant protection, HSP70 responses, and TLR signaling in cerebral ischemia remains insufficiently characterized.

This study was designed to evaluate the potential neuroprotective effects of the EDV‐loaded nanogel in a rat model of transient global ischemia (TGI) with particular emphasis on *Hsp70*, *Tlr4*, and *Tlr7* gene expression and to determine whether this nanogel‐based approach may provide a promising strategy for enhancing the therapeutic potential of EDV in ischemic brain injury.

## 2. Materials and Methods

### 2.1. Materials

All chemical solvents (isopropyl alcohol and ethanol) were purchased from Merck (Darmstadt, Germany). The substances used in RT‐PCR were purchased from multiple manufacturers, including TRIZOL reagent from Life Biolab (Heidelberg, Germany), loading buffer loading dye, EDTA, Tris base, and ladder from Sinaclon (Tehran, Iran). Primers were designed and developed in cooperation with PishgamBiotech (Tehran, Iran). The Primescript RT Reagent Kit and Master Mix Red were purchased from Takara Bio (Shiga, Japan) and Ampliqon (Odense, Denmark), respectively.

### 2.2. Animals

Fifty‐six male Wistar rats (200–220 g) were obtained from the Pasteur Institute of Iran (Tehran, Iran) and maintained under standard laboratory conditions, including 20°C–22°C temperature, relative humidity of 60*%* ± 5*%* with no limitation on access to food and water, and equal time day and night cycles (12:12‐h). All experimental procedures complied with the regulations of the University and National Institutes of Health (NIH) guidelines for the Care and Use of Laboratory Animals (Ethical Approval Number: IR.ZUMS.AEC.1402.010). The experimental design incorporated measures to minimize the number of animals used while maintaining appropriate standards of animal welfare. Finally, all animals were euthanized in a CO_2_ chamber, in compliance with established ethical standards.

### 2.3. TGI Model Induction

After anesthesia was induced by intraperitoneal (IP) administration of ketamine (80 mg/kg) and xylazine (8 mg/kg), the Wistar rats were positioned supine for the surgical procedure. The cervical region was shaved, disinfected with povidone–iodine (Betadine), and subsequently sterilized with 70% ethanol. A midline cervical incision was performed, and both the right and left common carotid arteries (CCAs) were carefully exposed and separated from the surrounding vagus nerves. TGI model was induced by applying vascular microclips to both CCAs. The common carotid was clamped on the neck site for 20 min, after which the clamp was removed and the site was stitched. The surgical incision was then irrigated and carefully sutured, and the animals were monitored during recovery under controlled conditions. Then, the animals first received EDV‐loaded nanogel or nanogel treatment 6 h after surgery. This method is frequently used to mimic the histopathological aspects of ischemic stroke, including neural hypoxia, inflammation, and neural damage [22–25].

### 2.4. Experiment Design

The 56 animals were randomly allocated to seven experimental groups (*n* = 7): (1) the control group was administrated normal saline; (2) the stroke group underwent a TGI induction procedure; (3) the stroke + NP group received GSH‐PMAA nanoparticles or nanogel 6 h after global ischemia induction; (4) the stroke + DEV(250 *μg*/kg) group received free form of DEV (250 *μg*/kg) in highest dose after 6 h global ischemia induction; (5–8) the treatment groups received EDV‐loaded nanogel at different relative doses to EDV (5, 20, 40, and 250 *μg*/kg). Twenty‐four hours following the final drug administrations, memory performance was evaluated using novel object recognition (NOR) test. Immediately following completion of the behavioral assessments, animals were euthanized in a CO_2_ chamber and following decapitation, the hippocampi were rapidly dissected on ice, snap‐frozen in liquid nitrogen and stored at −80°C until molecular analyses were performed.

### 2.5. Characterizations of Nanogel

Full characterization of nanogel product was done with different techniques based on previous study [25]. FTIR and HNMR analyses confirmed the successful synthesis of the nanogel. Dynamic light scattering revealed a mean particle size of approximately 199 nm, a polydispersity index of 0.282, and a zeta potential of −26.1 mV. Transmission electron microscopy showed predominantly spherical particles with no evident aggregation. The nanogel also provided sustained EDV release, with approximately 15% of the encapsulated drug released over 216 h. Notably, the formulation exhibited a high encapsulation efficiency (99.9%) and drug loading capacity (37.5%), supporting its stability as a controlled drug‐delivery system. Excellent physical stability of the EDV‐loaded nanogel was observed following 3 months storage in an aqueous solution [25].

### 2.6. Behavioral Test (NOR Test)

The main purpose of NOR is to investigate the tendency of rats to explore unfamiliar objects [26]. In the initial testing phase, each rat was individually placed in a square‐shaped Plexiglas chamber (70 × 70 × 30 cm). Two identical objects were placed at equal distances from the center of the box, and the rodents were allowed to explore them for 10 min. The next day, the rats were subsequently transferred to the previously described chamber, but one object was replaced by an unfamiliar one. The discrimination index was calculated as the ratio of the time spent investigating the novel object to the total exploration time for both objects.

### 2.7. Gene Expression Assay

Gene expression levels were quantified by real‐time quantitative PCR. Total RNA was isolated using TRIZOL reagent. In addition, 1 *μ*g of the extracted RNA was used for reverse transcription and cDNA production using SYBR Premix Ex Taq (Takara Bio, Japan) on a light‐cycler system (Roche Diagnostics, Mannheim, Germany). The cDNA then stored at −80°C for later polymerase chain reaction tests. For the PCR process, the list of utilized primers and their sequences are listed in Table 1. The temperature pattern consisted of one cycle of initial denaturation for 15 min at 95°C, followed by repeated extension/annealing for 40 separate cycles of 15 s each at 95 and 61°C, respectively. The extension phase had 40 cycles of 72°C for 30 s. *Gapdh* was used calculated by 2‐*ΔΔ*ct formula.

**Table 1:** The list of genes and their forward and reverse sequences.

Name	Sequence (5 ^′^ → 3 ^′^)	Genbank
*Hsp70*	GCTGCTGGTGCACGATTCTT	NM_001329896.1
	TTCGCAGGAAGGAAACACCA	
*Tlr4*	CCGCTCTGGCATCATCTTCA	NM_019178.2
	TCCCACTCGAGGTAGGTGTT	
*Tlr7*	TTCCCTGAATTCCGTGCCTC	NM_001097582.1
	CCAATCTCGCAGGGACAGTT	
*Gapdh*	ACTAACCCTGCGCTCCTG	NM_001256799.2
	CCCAATACGACCAAATCAGA	

### 2.8. Statistical Analyzes

Data are expressed as the mean ± SD. Statistical analyses were conducted using R studio program software. Comparisons between the groups were done analysis of one‐way analysis of variance (ANOVA) followed by Tukey′s multiple‐comparison post hoc test. *p* < 0.05 was considered statistically significant.

## 3. Results

### 3.1. The Discrimination Index Improved Following EDV‐Loaded Nanogel Treatment

**Figure 1:**
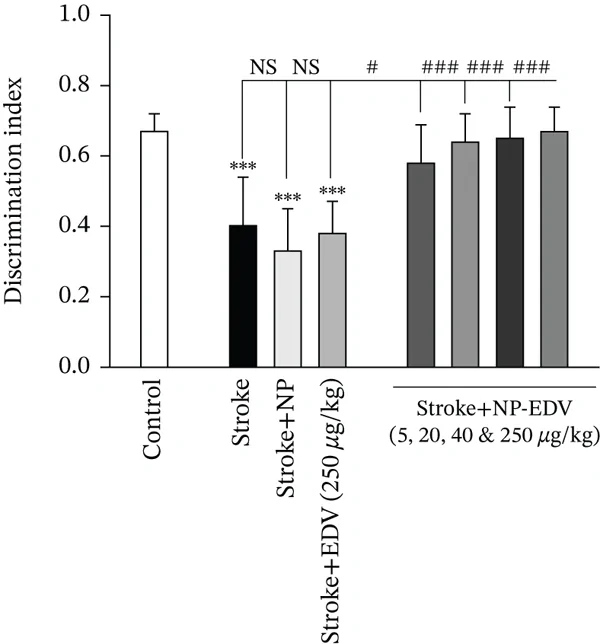
The effect of multiple doses of EDV‐loaded nanogel (5, 20, 40, and 250 *μ*g/kg) on discrimination index following novel object recognition test.  ^∗∗∗^ *p* < 0.001 compared with the control group. NS (nonsignificant), ^#^ *p* < 0.05 and ^###^ *p* < 0.001 compared with stroke rats. Values are expressed as the mean ± SD and were analyzed using one‐way ANOVA followed by the Tukey′s post hoc (*n* = 7 − 8).

As depicted in Figure 1, stroke induction significantly decreased the discrimination index compared with the control rats [*F*(7, 48) = 15.432;  ^∗∗∗^ *p* < 0.001]. Treatment with nanogel or free form of EDV in highest dose (250 *μg*/kg) in stroke rats did not improve the discrimination index when compared with the control rats (*p* > 0.05). Treatment with the EDV‐loaded nanogel significantly increased the discrimination index compared with the stroke group, with the strongest effects observed in EDV‐loaded nanogel at doses of 20, 40, and 250 *μg*/kg (^###^ *p* < 0.001).

### 3.2. The Expression of *Hsp70* Did Not Alter by Stroke or Treatment

**Figure 2:**
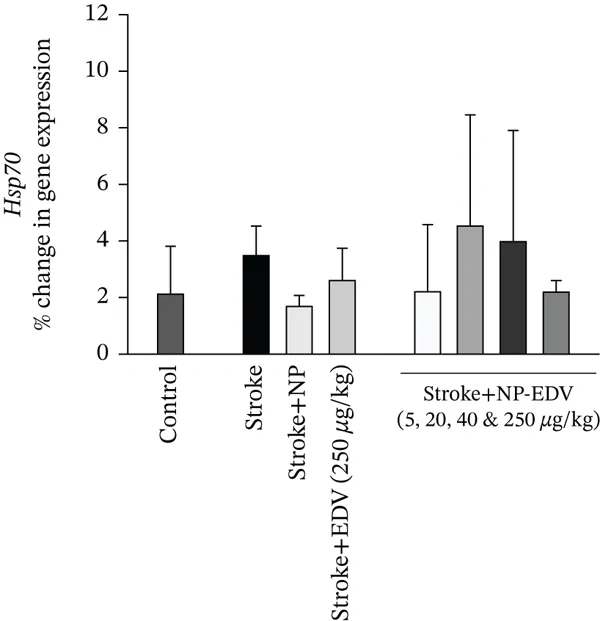
The effect of multiple doses of EDV‐loaded nanogel (5, 20, 40, and 250 *μg*/kg) on relative expression of *Hsp70*. Values are expressed as the mean ± SD and were analyzed using one‐way ANOVA followed by the Tukey′s post hoc (*n* = 3 − 4).

The relative expression of *Hsp70* did not significant change after stroke induction compared with control rats [*F*(7, 24) = 0.786; *p* = 0.606, Figure 2]. The expression level of *Hsp70* was not significantly altered by nanogel or EDV‐loaded nanogel or free form of EDV (250 *μg*/kg) treatment in stroke rats (*p* > 0.05).

### 3.3. The Expression of *Tlr4* Has Modulated by EDV‐Loaded Nanogel Treatment

**Figure 3:**
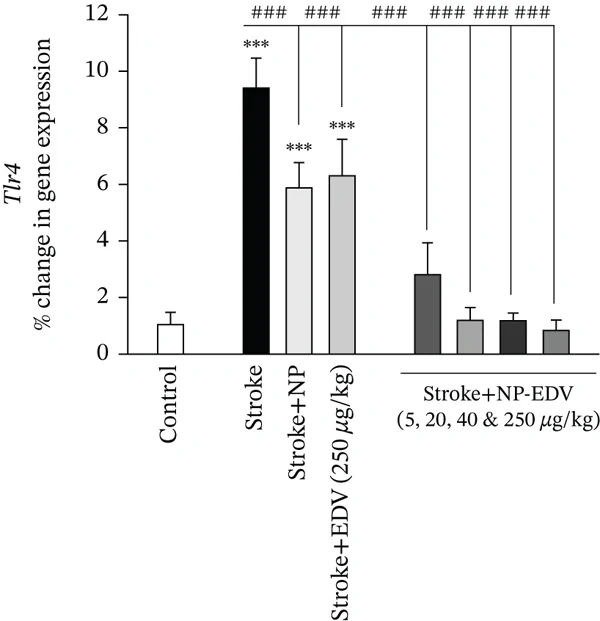
The effect of multiple doses of EDV‐loaded nanogel (5, 20, 40, and 250 *μg*/kg) on relative expression of Tlr4.  ^∗∗∗^ *p* < 0.001 compared with the control group and ^###^ *p* < 0.001 compared with stroke rats. Values are expressed as the mean ± SD and were analyzed using one‐way ANOVA followed by the Tukey′s post hoc (*n* = 3 − 4).

As depicted in Figure 3, *Tlr4* gene expression significantly increased following stroke induction rats in comparison with control rats [*F*(7, 24) = 61.938; *p* < 0.001]. Although this surge was almost halved after nanogel treatment (stroke+ nanogel) and free form of EDV (250 *μg*/kg) in the stroke rats, it reached a significant drop compared with the stroke group (^###^ *p* < 0.001), but still significant increase was observed compared with the control ( ^∗∗∗^ *p* < 0.001). Treatment with EDV‐loaded nanogel significantly halted the increase in *Tlr4* gene expression with a higher potential at applied doses of 20, 40, and 250 *μg*/kg compared with the stroke group (^###^ *p* < 0.001).

### 3.4. The Expression of *Tlr7* Has Modulated by EDV‐Loaded Nanogel Treatment

**Figure 4:**
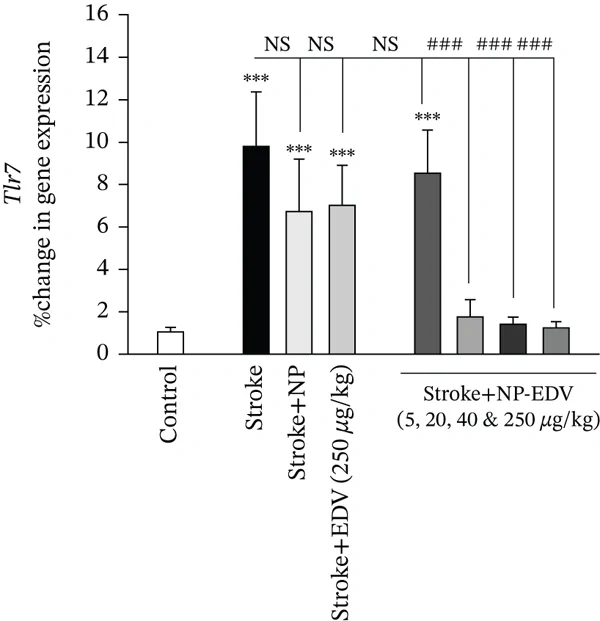
The effect of multiple doses of EDV‐loaded nanogel (5, 20, 40, and 250 *μg*/kg) on relative expression of Tlr7.  ^∗∗∗^ *p* < 0.001 compared with the control group. NS (nonsignificant), ^###^ *p* < 0.001 compared with stroke rats. Values are expressed as the mean ± SD and were analyzed using one‐way ANOVA followed by the Tukey′s post hoc (*n* = 3 − 4).

As presented in Figure 4, TGI rats significantly increased the relative expression of *Tlr7* compared with control group [*F*(7, 24) = 20.033;  ^∗∗∗^ *p* < 0.001]. Treatment with nanogel, free form of EDV in highest dose (250 *μg*/kg) in stroke induction rats, and stroke+5 *μg*/kg EDV‐loaded nanogel did not significantly change compared with the stroke group (*p* > 0.05). In contrast, EDV‐loaded nanogel treatment at doses of 20, 40, and 250 *μg*/kg in stroke rats significantly dropped *Tlr7* gene expression compared with the stroke group (^###^ *p* < 0.001).

## 4. Discussion

This study evaluated the neuroprotective effects and potential molecular mechanisms of EDV‐loaded nanogel in a rat model of TGI. Overall, treatment with EDV‐loaded nanogel improved recognition memory and modulated *Tlr4* and *Tlr7* expression, whereas *Hsp70* expression was not significantly altered at the selected postischemic time point. These findings, together with our previous evidence of enhanced brain delivery, sustained EDV release, reduced oxidative damage, and improved histopathological outcomes with the same formulation [25], support the potential of EDV‐loaded nanogel as a brain‐directed EDV delivery platform.

The cognitive improvement observed in NOR test suggests that EDV‐loaded nanogel can preserve hippocampus‐dependent recognition memory following cerebral ischemia. Cerebral ischemia disrupts cognitive function through interconnected mechanisms involving oxidative stress, neuro‐inflammation, mitochondrial dysfunction, and neuronal injury. In the present study, EDV‐loaded nanogel improved recognition memory at the tested doses, consistent with our previous findings [25]. Although beneficial effects of free EDV on spatial and episodic memory have also been reported [27–29], differences in animal models, doses, administration routes, treatment schedules, and behavioral paradigms preclude direct quantitative comparisons. Nevertheless, the cognitive benefit observed here is consistent with the previously demonstrated antioxidant and histopathological effects of EDV‐loaded nanogel and may reflect improved cerebral availability of EDV following nanogel‐mediated delivery.

The therapeutic rationale for EDV‐loaded nanogel is based on the complementary properties of EDV and the nanogel carrier [25]. EDV is a potent free‐radical scavenger that can attenuate lipid peroxidation and oxidative injury, whereas incorporation into a GSH‐functionalized nanogel provides controlled drug release and may enhance brain‐directed delivery while contributing additional antioxidant capacity [27–29]. In our previous study, the same formulation reduced malondialdehyde and protein carbonyl levels and restored endogenous GSH and FRAP in ischemic brain tissue [25]. These results suggest that the treatment with EDV‐loaded nanogel attenuated oxidative stress while partially recovering the endogenous antioxidant defense system. The present behavioral and molecular findings extend this evidence by suggesting that the effects of EDV‐loaded nanogel may involve not only redox regulation but also modulation of innate immune signaling. Consistent with this possibility, EDV‐loaded nanogel significantly modulated *Tlr4* expression, which is an important mediator of sterile neuro‐inflammation following cerebral ischemia and can promote microglial activation [30, 31]. Decrease of *Tlr4* gene expression following GSH‐PMAA‐EDV treatment suggests attenuation of a component of postischemic innate immune activation. This observation is compatible with previous evidence that EDV can suppress TLR4‐associated inflammatory pathways, including NF‐*κ*B/NLRP3 signaling [32, 33]. However, these results should be regarded as evidence of changes in *Tlr4* gene expression rather than direct suppression of TLR4‐mediated signaling.

The effects on *Tlr7* provide an additional and potentially novel aspect of the present findings. TLR7 is an intracellular pattern‐recognition receptor that can activate MyD88‐dependent inflammatory signaling, although its effects may depend on cellular context and the stage of injury [15, 34–36]. Our data showed that EDV‐loaded nanogel restored *Tlr7* gene expression toward control levels at 20, 40, and 250 *μ*g/kg of EDV. The concurrent modulation of *Tlr4* and *Tlr7* may therefore indicate broader regulation of postischemic innate immune responses rather than simple suppression of inflammatory signaling. Because the contribution of TLR7 to ischemic brain injury remains less well characterized than that of TLR4, these findings should be considered preliminary and validated by western‐blotting analyses.

HSP70 represents another component of the cellular stress response that may interact with oxidative and inflammatory pathways after cerebral ischemia [20, 36–38]. In the present study, *Hsp70* mRNA expression did no significant difference between treatments between EDV‐loaded nanogel in stroke animal models and stroke or control rats. This finding does not exclude a role for HSP70 in the neuroprotective response because its expression is strongly dependent on the timing and severity of cellular stress. One important explanation is the timing of tissue collection. Importantly, HSP70 induction is generally more prominent during the early phase of postischemic period [20, 37], whereas gene expression in the present study was assessed on Day 7. Moreover, changes in *Hsp70* transcription do not necessarily reflect changes in HSP70 protein level. Thus, the absence of a significant Day‐7 transcriptional response should be interpreted cautiously. Future studies incorporating multiple early and late time points and simultaneous assessment of HSP70 mRNA and protein will be necessary to clarify its contribution. The present findings should also be considered in the context of our previous characterization of GSH‐PMAA‐EDV.

The formulation demonstrated favorable physicochemical characteristics, high drug encapsulation, sustained EDV release, and enhanced brain delivery, together with reduced oxidative and histopathological injury after cerebral ischemia [25]. Other nanotechnology‐based approaches, including liposomes, polymeric nanoparticles, intranasal hydrogels, and exosome–liposome hybrid systems, have also been investigated to improve EDV delivery and brain exposure [38–41]. Although EDV‐loaded nanogel shows potential advantages, direct comparisons with free EDV and alternative nanocarriers are required to establish its relative therapeutic efficacy.

From a translational perspective, the EDV‐loaded nanogel may offer advantages over free EDV, including sustained drug release, high drug encapsulation, and enhanced brain delivery. Our previous study showed improved cognitive performance, increased antioxidant capacity, and reduced oxidative damage with the same formulation, supporting its potential to enhance EDV availability in the brain [25]. However, the mechanisms underlying BBB transport and the specific contribution of surface‐conjugated glutathione remain unclear. Further pharmacokinetic, bio‐distribution, BBB transport, and dose‐optimization studies are therefore required. Although no apparent histopathological toxicity was observed at the tested doses in our previous study [25], this provides only preliminary evidence of short‐term tolerability. Comprehensive long‐term safety studies addressing systemic toxicity, polymer degradation and clearance, tissue accumulation, organ function, and immunogenicity, including evaluation in both sexes and larger animal models, are warranted before clinical translation.

Several limitations should be acknowledged. First, the present study primarily assessed *Tlr4*, *Tlr7*, and *Hsp70* at the transcriptional level without corresponding protein or functional measurements. Second, inflammatory cytokines and downstream signaling components, including NF‐*κ*B and NLRP3, were not quantified. Third, behavioral assessment was limited primarily to recognition memory, and additional motor, sensorimotor, and neurological outcomes would provide a more comprehensive assessment of functional recovery. Finally, infarct or lesion volume, neuronal survival, BBB integrity, and microglial and astrocytic activation were not directly evaluated. These limitations restrict mechanistic interpretation and should be addressed in future studies.

Overall, EDV‐loaded nanogel improved postischemic recognition memory and modulated *Tlr4* and *Tlr7* expression, extending our previous evidence of antioxidant and histopathological protection by the same formulation [25]. The findings suggest that the neuroprotective effects of EDV‐loaded nanogel may involve coordinated regulation of oxidative stress and innate immune responses. However, validation at the protein and functional levels, together with temporal profiling and comprehensive pharmacokinetic, bio‐distribution, and safety studies, is required to establish the underlying mechanisms and translational potential of this nanogel‐based EDV delivery strategy.

## Author Contributions

**Leila Nasehi**: conceptualization, study design, data curation, investigation, methodology, supervision, project administration, writing—original draft. **Salar Jahangiri**: data curation, formal analysis, investigation, visualization, writing—original draft. **Soroush Bijani:** data curation, formal analysis, investigation, visualization, writing—original draft. **Mir-Jamal Hosseini:** conceptualization, study design, funding acquisition, project administration, methodology, supervision, writing—review and editing.

## Funding

This study was supported by Zanjan University of Medical Sciences (A‐12‐769‐60).

## Disclosure

All the authors have read, approved, and made substantial contributions for the manuscript.

## Consent

The authors have nothing to report.

## Conflicts of Interest

The authors declare no conflicts of interest.

## Data Availability

The data that support the findings of this study are available on request from the corresponding author. The data are not publicly available due to privacy or ethical restrictions.
